# Event-Related Potential Measures of Attention Capture in Adolescent Inpatients With Acute Suicidal Behavior

**DOI:** 10.3389/fpsyt.2018.00085

**Published:** 2018-03-19

**Authors:** Paniz Tavakoli, Addo Boafo, Allyson Dale, Rebecca Robillard, Stephanie L. Greenham, Kenneth Campbell

**Affiliations:** ^1^Children’s Hospital of Eastern Ontario Research Institute, Ottawa, ON, Canada; ^2^Department of Psychiatry, University of Ottawa, Ottawa, ON, Canada; ^3^Children’s Hospital of Eastern Ontario, Ottawa, ON, Canada; ^4^School of Psychology, University of Ottawa, Ottawa, ON, Canada; ^5^University of Ottawa Institute of Mental Health Research, Ottawa, ON, Canada

**Keywords:** suicidality, executive functions, attention capture, event-related potentials, P3a

## Abstract

Impaired executive functions, modulated by the frontal lobes, have been suggested to be associated with suicidal behavior. The present study examines one of these executive functions, attentional control, maintaining attention to the task-at-hand. A group of inpatient adolescents with acute suicidal behavior and healthy controls were studied using a passively presented auditory optimal paradigm. This “optimal” paradigm consisted of a series of frequently presented homogenous pure tone “standards” and different “deviants,” constructed by changing one or more features of the standard. The optimal paradigm has been shown to be a more time-efficient replacement to the traditional oddball paradigm, which makes it suitable for use in clinical populations. The extent of processing of these “to-be-ignored” auditory stimuli was measured by recording event-related potentials (ERPs). The P3a ERP component is thought to reflect processes associated with the capturing of attention. Rare and novel stimuli may result in an executive decision to switch attention away from the current cognitive task and toward a probe of the potentially more relevant “interrupting” auditory input. On the other hand, stimuli that are quite similar to the standard should not elicit P3a. The P3a has been shown to be larger in immature brains in early compared to later adolescence. An overall enhanced P3a was observed in the suicidal group. The P3a was larger in this group for both the environmental sound and white noise deviants, although only the environmental sound P3a attained significance. Other deviants representing only a small change from the standard did not elicit a P3a in healthy controls. They did elicit a small P3a in the suicidal group. These findings suggest a lowered threshold for the triggering of the involuntary switch of attention in these patients, which may play a role in their reported distractibility. The enhanced P3a is also suggestive of an immature frontal central executive and may provide a promising marker for early identification of some of the risk factors for some of the cognitive difficulties linked to suicidality.

## Introduction

Adolescence is a phase of life associated with possible increased risks for dangerous behaviors. The World Health Organization indicates that suicide is the fifteenth leading cause of death worldwide but represents the second leading cause of death among 10–24 years old (1). Mood disorders are among the top mental health illnesses that increase the risk of suicidal behavior (2). Research has shown that internal conflicts as well as feelings of depression and anxiety can result in suicidal behavior (3, 4).

Attempted suicide may be a different phenomenon in adolescents than in adults. This is particularly true considering that adolescence is a critical time for brain maturation and the development of cognitive and emotional personalities. Adolescents with suicidal behavior often exhibit concentration deficits that can be displayed as increased distractibility (5–7). These deficits are often increased with the presence of suicide attempts. Furthermore, distractibility in these individuals may be caused by their high emotional sensitivity to external stimuli, particularly negative emotional cues [for reviews, see Ref. (8, 9)]. This emotional sensitivity may in turn predispose these individuals to self-harming and suicidal behavior (10, 11). The present study aims to determine whether information processing commonly associated with distractibility and attention shifts are affected in adolescents with an acute risk of suicidal behavior.

Imaging studies have provided evidence that a number of regions of the frontal lobe are implicated in suicidal behavior (12–18). Marzuk et al. (19) was among one of the first to suggest that impaired executive functions, modulated by the frontal lobes, may be associated with suicidal ideation, regardless of a history of suicide attempts. A number of neuropsychological and cognitive studies have now provided support for this claim [see Ref. (20–22) for reviews]. There is much debate whether the frontal lobes modulate a single unitary or “central” executive function or whether there are many interrelated and fragmented functions (23, 24). Stuss et al. (25) have identified different subregions within the frontal lobes that reflect these separate, fragmented functions. In this regard, research has shown that suicidal ideation and actual suicide attempt are associated with many different deficits in executive functions (21, 26–31). The deficits in executive tasks that have been identified vary among studies and have not always been replicated. Part of this inconsistency can be explained by factors such as the diversity in patient samples (suicide ideation versus attempt), the nature and time (acute, recent, remote) of the attempts, comorbid disorders and medications (21). Keilp et al. (29, 32) however emphasize that common to many studies is a specific executive deficit, attentional control. This may be observed in an inability to sustain attention for long periods of time.

The need to sustain active attention is critically important to many higher aspects of cognition. Almost all neuropsychological and cognitive tasks do require attention to be maintained for the duration of the task. It is, thus, possible that reports of a dysfunction on a wide number of executive functions may be confounded by this underlying inability to sustain sufficient attention to maintain optimal performance. The present study examines an executive function that operates passively, independent of active attention. While an individual is actively engaged in a cognitive task, certain potentially highly relevant auditory events occurring outside of the current focus attention may result in a switch of attention away from the cognitive task and toward the auditory stimulation. Such control of attentional resources is called passive attention (33). In this regard, Keilp et al. (28) note that deficits in attentional control do not involve all aspects of attention but rather are more specific to this interference processing. A critical executive function is indeed to allocate attentional resources and to maintain attention to highly relevant tasks. Nevertheless, the system is fluid and flexible. For survival purposes, we must be able to detect, and act upon, potentially highly relevant information occurring outside of the current focus of attention. Nevertheless, most stimulus input that bombards the sensory receptors does in fact turn out to be irrelevant. A consequence of these interruptions is deterioration in performance of the current cognitive tasks as a result of attention being switched away. This is called “distraction.” A delicate balance must thus be established that limits interruption of current cognitive demands to only very rare and highly relevant stimulus input. The Keilp et al. (28) interference hypothesis suggests that suicidal behavior may be marked by a too frequent interruption of the central executive.

It is, of course, difficult to design experimental studies to measure the extent of processing of information occurring outside the current focus of attention. The participant could be asked to detect, by button pressing, certain stimuli occurring in an unattended sensory modality. However, active attention is then being directed to that modality, bringing it into the focus of attention. Auditory event-related potentials (ERPs) allow researchers to monitor the extent of information processing of stimulus input occurring outside the focus of attention. ERPs are the minute changes in the ongoing electrical activity of the brain (the “EEG”) that are elicited by external stimuli or internal psychological events. ERPs consist of a series of negative-voltage and positive-voltage “components,” reflecting different aspects of information processing. Auditory stimuli are often used in the study of attention capture. This is because we hear over 360° and all auditory stimuli, whether attended or ignored, are processed to a certain extent. Visual stimuli that are not presented within the participant’s visual field, on the other hand, do not activate retinal cells and are thus not processed in the visual system.

Many auditory ERP studies of attention capture employ a so-called auditory “oddball” paradigm. The participant is presented with a rapid presentation of a frequently occurring homogenous “standard” stimulus. Occasionally, a feature of the standard stimulus is changed to form a rarely occurring “deviant” stimulus. The participant is often asked to ignore the auditory sequence of stimuli while attending to another, often visual, task. The auditory ERPs are thus elicited passively, independent of active attention. Such processing is thus said to be preattentive, or preconscious. The standard stimulus elicits an obligatory negative-going component, “N1,” peaking at about 100 ms after stimulus onset. This is followed by a positivity known as the “P2,” occurring at about 180–200 ms. Deviant stimuli also elicit the same N1-P2. In addition, it also elicits another negative ERP component, the mismatch negativity [MMN; (34, 35)]. The MMN occurs at about 100–200 ms following the onset of the stimulus depending on the extent of change from the standard stimulus. The MMN’s voltage is largest over frontocentral areas of the scalp, and it inverts in polarity (recorded as a positive potential) at the mastoids. The MMN is associated with a preconscious memory-based change detection system in which the features of the incoming auditory stimulus are compared against the features of the preceding stimuli stored in sensory memory. If a standard is now presented, the features of the incoming stimulus match those stored in a sensory memory; memory is then improved, but further processing ceases. The individual would thus not be conscious of this input. If the features do not match those stored in sensory memory, change is detected. A more recent model suggests that the MMN is elicited by the detection of deviance whenever an external auditory event does not match the brain’s prediction of environmental regularities (36–39), the frequently presented standard stimulus, in this case, representing a pattern of regularity. Importantly, the change detection system operates automatically and independently of attention. Thus, the MMN is elicited even if the participant is not attending to the auditory channel in which the deviant occurs and regardless of task demands in which the participants is engaged (40–42). Highly novel deviants might elicit both a larger N1 than the standard stimuli, but also an MMN. This observed is often referred to as a deviant-related negativity (DRN) as it is not a true MMN. It represents a combined negative potential, both spatially and temporally, of the N1 and MMN. In this article, for consistency, the negativity following deviants will be described as a DRN.

The output of the change detector system is proportional to the extent of change between standard and deviant. If this output is large enough, it will send a trigger to the central executive, resulting in a passive switch of attention away from current cognitive demands and toward a probing of the “interrupting” auditory modality. The content of the auditory modality then becomes available to consciousness. This process has been associated with a later 200–250 ms positivity, maximum over centrofrontal areas of the scalp, the P3a (43). There is currently some debate about whether the P3a reflects the actual switch of attention toward incoming auditory stimuli or is a precursor process that may lead to conscious awareness [for reviews, see Ref. (44, 45)]. The current understanding is that the presence of the P3a at least reflects higher-level processing, such as the evaluation of events as being significant (46–48). Several studies have reliably shown that the presentation of certain irrelevant auditory deviants that elicit a P3a will cause a distraction away from other primary tasks as evidenced by increased reaction times and/or decreased accuracy of detection (49–52). It is important to note that while almost any perceptible acoustic change will elicit an MMN, only a few of these auditory stimuli will also elicit a P3a. The P3a is elicited by only those stimuli that signal a large extent of change. In the Näätänen model (34, 35), the threshold at which the central executive is interrupted is thought to be flexible. In certain disorders, it may be very low, resulting in abnormally frequent interruption while in other disorders, very high, resulting in an inability to detect potentially relevant events occurring outside of the focus of attention.

It has long been known that prefrontal cortical regions play an important role in the orienting of attention (53). The prefrontal cortex is however not fully mature until late adolescence [see (54, 55) for reviews]. It has been suggested that the P3a is associated with a complex network of brain regions involved in processing and evaluating novel information. These regions include the prefrontal cortex, anterior cingulate cortex, and the hippocampus (56–58). A limited number of studies have examined attention capture and the involuntary switching of attention, as measured by the P3a, in adolescents. There is evidence of an increased susceptibility to task-irrelevant information in children and younger adolescents compared to older adolescents and adults (45, 52, 59–65). It is thus possible that the threshold for interruption of the central executive is lower in younger participants.

Few studies have examined the P3a in association with suicidal behavior. Similarly, a limited number of studies have been carried out in patients with major depressive disorder (MDD), and the results are inconsistent (66–70). In adults, the amplitude of the P3a appears to be reduced in patients with MDD compared to healthy controls (66–68, 70). On the other hand, Lepistö et al. (71) examined the P3a in 10- to 13-year-old children with MDD and observed an enhanced P3a in response to the rarely occurring deviant stimuli, in contrast to the studies showing a reduced P3a in adults. Additionally, although Jandl et al. (69) did not directly compare P3a amplitudes between adult depressed suicide attempters and healthy controls, their figures show a slight enhancement of the P3a in the suicidal patients [Figure 2 in Ref. (69)]. These limited findings thus suggest a very different pattern of processing from adolescent to adult MDD. Of course, differences in methodology and choice of types of deviant stimuli limit this conclusion. It is possible that the type of deviant stimuli used can differentially affect the interruption of the central executive in depressed/suicidal adolescents and healthy controls. Adolescents with suicidal behavior may exhibit a frequent occurrence of a P3a to deviant stimuli that do not elicit a P3a in controls. To determine whether this is the case requires the presentation of several different deviants, varying in extent of change from the standard.

A problem with ERP methodology is that the “signal” of interest (the P3a in this case) is embedded within the much larger ongoing background EEG “noise.” The amplitude of the background EEG can be reduced through repeated presentation of the same stimulus and the averaging of these trials. The amplitude of the background EEG will decrease with the averaging procedure while the ERP signal should remain constant from one trial to the next. This procedure will decrease the signal-to-noise ratio. Nonetheless, in order for the ERP signal to emerge from the background EEG noise, numerous stimulus repetitions must be presented. An oddball sequence will often last from 10 to 15 min to permit a sufficiently large number of stimuli to be presented. Researchers often replicate their data, in which case the sequence will need to be repeated a second time. Furthermore, if several oddball sequences are to be presented to examine the P3a to numerous deviants, testing times could be very long indeed, exceeding 2 h. Such a long testing time may not be feasible in clinical populations.

A newer multifeature optimal paradigm (72) reduces testing time dramatically because several different deviants are presented within a single sequence. In this paradigm, standard and deviant stimuli alternate. Thus, the overall probability of occurrence of each is 0.50. However, several different types of deviants are presented. In the original multifeature paradigm, each deviant represented a change in a different feature from the standard (for example, its frequency, duration, intensity, etc.). Thus, if five different deviants are presented, even though the overall probability of occurrence of deviance was quite high (0.50), the probability of occurrence of a specific deviant was low (0.10). While a single feature of the deviant does differ from that of the standard, all other features are shared. The optimal paradigm was created with the assumption that the deviant stimuli will strengthen the memory trace of the standard in regards to the stimulus features they share. The MMNs that were elicited when an optimal paradigm was run were very similar to the MMNs elicited when separate oddball paradigms were run for each deviant (72). Recently, Tavakoli and Campbell (73) studied whether an optimal paradigm could also be used for the study of the P3a in young healthy adults. They noted that only certain deviants, white noise and novel environmental sounds, could elicit a P3a within an oddball paradigm. Other types of deviants did not. Very similar findings were found when the same deviants were used within a single optimal sequence. The advantage the multifeature optimal paradigm is that it of course significantly reduces testing times and is thus particularly suited for use with clinical populations.

Very few ERP studies have been run with suicidal populations. The present study examines processing related to a critical executive function, the capturing of attention. Most tests of executive function require the maintenance of attention for relatively long periods of time. The capture of attention is, however, a relatively passive process not requiring active attention. There is evidence that frequent interruptions of the central executive by irrelevant input are a risk factor for suicidal behavior. The present study will record ERPs during the presentation of a multifeature optimal paradigm to determine whether adolescents with suicidal behavior are more likely to show a P3a, reflecting attention capture processing.

## Materials and Methods

### Participants

The study participants were 12 (10 females) adolescent psychiatric in-patients admitted for acute risk of suicide and 12 (10 females) age and gender matched healthy controls. Adolescents ranged in age from 13 to 17 years (mean = 14.9; SD = 1.2). Patients were recruited after coming to the Emergency Unit and then being admitted to the inpatient psychiatric unit at the Children’s Hospital of Eastern Ontario. None of participants had any reported a history of hearing or neurological disorders. Written informed consent was obtained from all participants, and parents when necessary, prior to the start of the study. Participants received an honorarium for their participation. The study was approved by both the University of Ottawa’s Health Sciences and Science Research Ethics Board and the Children’s Hospital of Eastern Ontario’s Research Ethics Board. The study was conducted according to the Canadian Tri-Council guidelines (Medical, Natural, and Social Sciences) on ethical conduct involving human subjects. These guidelines are similar to those used conducted with the Declaration of Helsinki.

#### Medications

A requirement was that potential in-patient participants were not being treated using benzodiazepines prior to the start of the study. All patients were treated with medication, including antidepressants [selective serotonin reuptake inhibitors (SSRIs) and selective serotonin and norepinephrine reuptake inhibitors] and/or atypical antipsychotics. Three patients reported sleep disturbances and were treated using melatonin. Studies have shown that the MMN is not significantly modulated by antidepressant (74, 75) or atypical antipsychotic (74, 76–80) medications. Very few studies have directly examined the effects of these medications on the amplitude of the P3a. Rydkjaer et al. (74) found no significant differences in the amplitude of the MMN and P3a among those with and without SSRI antidepressant use and also among those with and without antipsychotic use.

### Psychological Assessment

The severity of depression symptoms was assessed using the Children’s Depression Inventory-2 [CDI-2; (81)], a commonly used self-report rating inventory that includes 27 items. The 27 items are grouped into two major factor, each comprised of two subscales assessing emotional problems (including negative mood/physical symptoms and negative self-esteem) and functional problems (including ineffectiveness and interpersonal problems). Patients had a mean CDI-2 total score of 24.88 (SD = 9.49). Controls had a mean CDI-2 total score of 4.1 (SD = 2.93). The presence and severity of suicidal symptoms were assessed using the Suicidal Behaviors Questionnaire-Revised [SBQ-R; (82)], a brief 4-item, self-report questionnaire measuring different dimensions of suicidality: (1) lifetime suicide ideation and suicide attempt, (2) frequency of suicide ideation over the past 12 months, (3) threat of suicidal behavior, and (4) the likelihood of suicidal behavior. Patients had a mean SBQ-R score of 13.75 (SD = 2.37). Controls had a mean score of 3.5 (SD = 0.71). The CDI-2 and SBQ-R scores did significantly differ between patients and controls (*p* < 0.05 in both cases).

### Neurophysiological Recording

EEG and electrooculography (EOG) activity were recorded using Grass gold-cup electrodes, filled with electrolytic paste, and affixed to the skin by surgical tape and to the scalp by gauze. Brain Products BrainAmp amplifiers and Recorder software were used for the recording of the physiological signals. The EEG was recorded from 11 electrodes across frontal, central, parietal, and occipital sites (F3, Fz, F4, C3, Cz, C4, P3, Pz, P4, O1, O2) according to the 10/20 system of electrode placement. Two additional electrodes were placed on the left and right mastoids (M1 and M2). Vertical EOG was recorded from electrodes placed at the supra-orbital and infra-orbital ridges of the left eye. A horizontal EOG was recorded from electrodes placed at the outer canthus of each eye. The nose served as a reference for all channels, including the EOG channels. Inter-electrode impedances were kept below 5 kΩ. The high-frequency filter was set at 75 Hz and the time constant was set at 2 s. The physiological data were digitized continuously at a 500 Hz sampling rate and stored on hard disk for later analyses.

### Procedure and Stimuli

Auditory stimuli were presented monaurally to the right ear using EAR 3A insert earphones while participants watched a silent, subtitled movie of their choice. The auditory stimuli were thus irrelevant and participants were asked to ignore them. A multifeature auditory optimal paradigm was presented. This permitted the presentation of six different deviant stimuli within a singe sequence. The participants was presented with a sequence constructed such that every other stimulus was a 80 dB SPL 1,000 Hz “standard” pure tone (*p* = 0.5) and every other was one of six deviants (each with a *p* = 0.08). Deviants in the optimal sequence included (a) a 90 dB SPL increment pure tone, (b) a 60 dB SPL decrement pure tone, (c) an 80 dB peak SPL white noise burst, (d) different environmental sounds (with an average intensity of 80 dB SPL), (e) a higher frequency, 1,100 Hz, pure tone, and (f) a shorter duration, 100 ms, pure tone. All stimuli had a duration of 200 ms and a rise-and-fall time of 5 ms, with the exception of the duration deviant. Table 1 lists the properties of the various auditory stimuli. The deviants were pseudorandomized so that in an array of six deviants, each deviant was presented once and the same deviant was never presented twice in a row. Thus, while every other stimulus was a deviant, the participants could not predict which specific deviant would be presented. A different environmental sound was presented on each trial so that none of the environmental sounds were repeated. The environmental sounds were downloaded from the New York Psychiatric Institute [described in Ref. (83)]. Their duration was however manipulated to be the same 200 ms as the other stimuli. The environmental sounds consisted of six different categories of stimuli including, animal sounds (e.g., dog, cat, frog), bird sounds, human-produced sounds (e.g., laughter, coughing, sneezing, hiccup), musical instruments (e.g., piano, violin, guitar), sounds within daily environments (e.g., water dripping, drilling, car, video games), and mechanically produced sounds. The first 10 tones in the sequence consisted of only standards in order to establish a memory trace for the standard stimulus. The stimulus onset asynchrony (onset-to-onset) was 600 ms. A total of 932 stimuli were presented in a single sequence, consisting of 472 trials of standards and 77 trials of each deviant. A sequence thus lasted about 9.5 min. Two blocks of auditory stimuli were presented to both patients and controls. A brief 5-min rest period was given between blocks.

**Table 1:** The intensity, frequency, duration, and probability of the standard stimulus and the six deviant stimuli in the optimal paradigm.

Stimulus type	Intensity	Frequency	Duration	Probability
Standard	80 dB SPL	1,000 Hz	200 ms	0.50
Deviants				
Frequency	80 dB SPL	*1,100* *Hz*	200 ms	0.08
Increment	*90* *dB SPL*	1,000 Hz	200 ms	0.08
Decrement	*60* *dB SPL*	1,000 Hz	200 ms	0.08
Duration	80 dB SPL	1,000 Hz	*100* *ms*	0.08
White noise	~*80 dB SPL*	*Random*	200 ms	0.08
Environmental sounds	~*80 dB SPL*	*Mixed*	200 ms	0.08

*Information in italics represents the feature of the deviant that has been changed*.

### ERP Analysis

The data were reconstructed using Brain Products’ Analyzer2 software. The continuous EEG data was band-pass filtered between 0.5 and 20 Hz (24 dB/octave slope). A vertical EOG channel was computed by subtracting activity recorded at supraorbital and infraorbital ridges of the left eye. A horizontal EOG channel was computed by subtracting activity recorded at the outer canthus of each eye. Independent Component Analysis (84, 85) was used to identify eye movement and blink artifacts that were statistically independent of the EEG activity. These were then partialed out from the EEG trace. The continuous data were subsequently reconstructed into discrete single trial 700 ms segments, beginning 100 ms before stimulus onset and then baseline corrected. The prestimulus period for the environmental sound deviants was not stable and varied between the two groups therefore a −50 to 50 ms parastimulus baseline was applied. Segments in which EEG activity exceeded ±100 μV relative to the baseline were excluded from further analyses. No more than 5% of total trials were rejected from further analyses per participant. There was no variation in the rejection of trials across deviants. The single trials were then sorted and averaged on the basis of stimulus type (standard and six deviants) and electrode site.

### Quantification and Statistical Analyses

**Figure 1:**
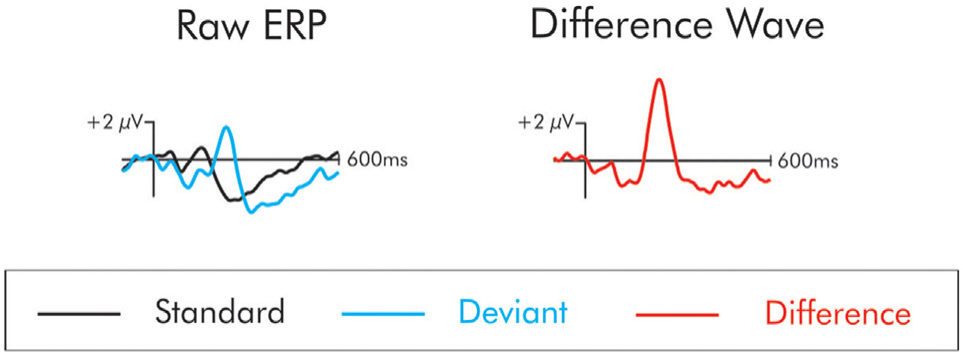
The “raw” event-related potentials (ERPs) following the presentation of the standard and deviant are traced on the left. The difference wave on the right is constructed by subtracting the raw standard from the deviant waveforms. The subtraction process removes the commonalities in processing between the standard and the deviant leaving only processing unique to the deviant. In the difference wave, a small negativity is apparent at about 100 ms. This is the mismatch negativity/deviant-related negativity. This deviant also elicits a large positivity at about 225 ms. This is the P3a.

The ERP waveform time-locked to the deviant stimuli elicited a series of positive- and negative-going components that were not apparent in the waveform following the standard stimulus. These components are best observed in a difference wave computed by subtracting, point-by-point, the standard from the deviant waveforms at each electrode site. This process removes the commonalities in processing between the standard and the deviant, leaving only processing unique to the deviant. The subtraction process is illustrated in Figure 1. From this difference wave, the DRN and P3a were initially identified using the grand averaged data (the average of all subjects’ averages) separately for patients and controls. The DRN and P3a were measured with respect to the prestimulus zero voltage baseline. They were quantified for each individual participant using the mean of all the data points that were within ±25 ms of the peak in amplitude that was identified in the grand average.

Previous studies have indicated that not all deviants will elicit a P3a. The statistical analysis of absent ERP components with the usual analyses of variance (ANOVA) procedure is problematic. This is because the observation of a significant amplitude difference with, for example, the P3a between participant groups cannot be used as evidence that a specific deviant did in fact elicit this component for a given group. It must be first demonstrated that the deviants elicited significant ERP components. Thus, confidence intervals were computed for the P3a. When the lower limit of the interval was significantly greater than 0 µV (i.e., in a positive direction), it was considered to be a significant positivity. The procedure was run at Cz where the P3a tends to be at maximum amplitude. Because a positive directionality was predicted, one-tailed tests of significance (*p* < 0.05) were applied to the confidence intervals. To restrict the likelihood of chance findings, the positivity had to conform to the usual latency (180–350 ms) and scalp distribution (centrofrontal maximum) of the P3a.

Electrode sites were grouped into regions of interest (ROIs), to include nine electrode sites where the ERP components have been quantified in previous studies. The ROIs allowed for an analysis of an anterior–posterior and an interhemisphere factor. Specifically for the anterior–posterior electrode factor, three electrodes for frontal (F3, Fz, F4), central (C3, Cz, C4), and parietal (P3, Pz, P4) sites were chosen for separate analysis. The DRN and P3a components were thus quantified at each of these sites within the latency range identified at Fz, and Cz, where their amplitude is largest. For the interhemisphere factor, three electrodes for left (e.g., F3), midline (e.g., Fz), and right (e.g., F4) sites were chosen for analysis.

The between-group differences in the amplitudes of the ERP components were tested using ANOVA. Specific details about the exact nature of each statistical analysis are reported in the “Results” section. Separate ANOVAs were conducted for the DRN and P3a. Significant main effects and interactions were followed up with least significant difference *post hoc* testing. For all statistical analyses, a Geisser-Greenhouse correction was used when appropriate (86).

## Results

### Standard ERP

**Figure 2:**
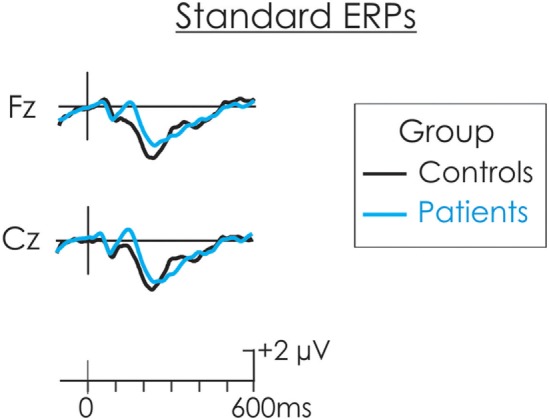
The event-related potential (ERP) waveform to the standard stimulus for both patients (blue) and controls (black). There were no significant differences in the amplitude of the N1 or P2 between patients and controls.

The DRN and P3a waveforms were calculated in the deviant-standard difference wave. As a result, an assumption is made that processing of the standard is similar for both groups and thus, whatever differences emerged were a result of differential processing of the deviant. The ERP waveform to the standard stimulus for both patients and controls in presented in Figure 2. This assumption was tested. The N1 and N2 were measured at Cz in the standard waveform as the mean of all data points within ±25 ms of the peak identified in the grand average. A *t*-test was run separately on the amplitude of the N1occurring at about 100 ms and P2 occurring t about 180 ms, between patients and controls. There was no significant difference in the amplitude of the N1 (*t* < 1) or P2, *t*(22) = 1.88, *p* > 0.05 between the two groups. An additional negativity at about 220 ms was also observed in the ERPs following the standard stimulus. There was no significant difference in the amplitude of this negativity between patients and controls (*t* < 1).

### Deviant-Related Negativity

**Figure 3:**
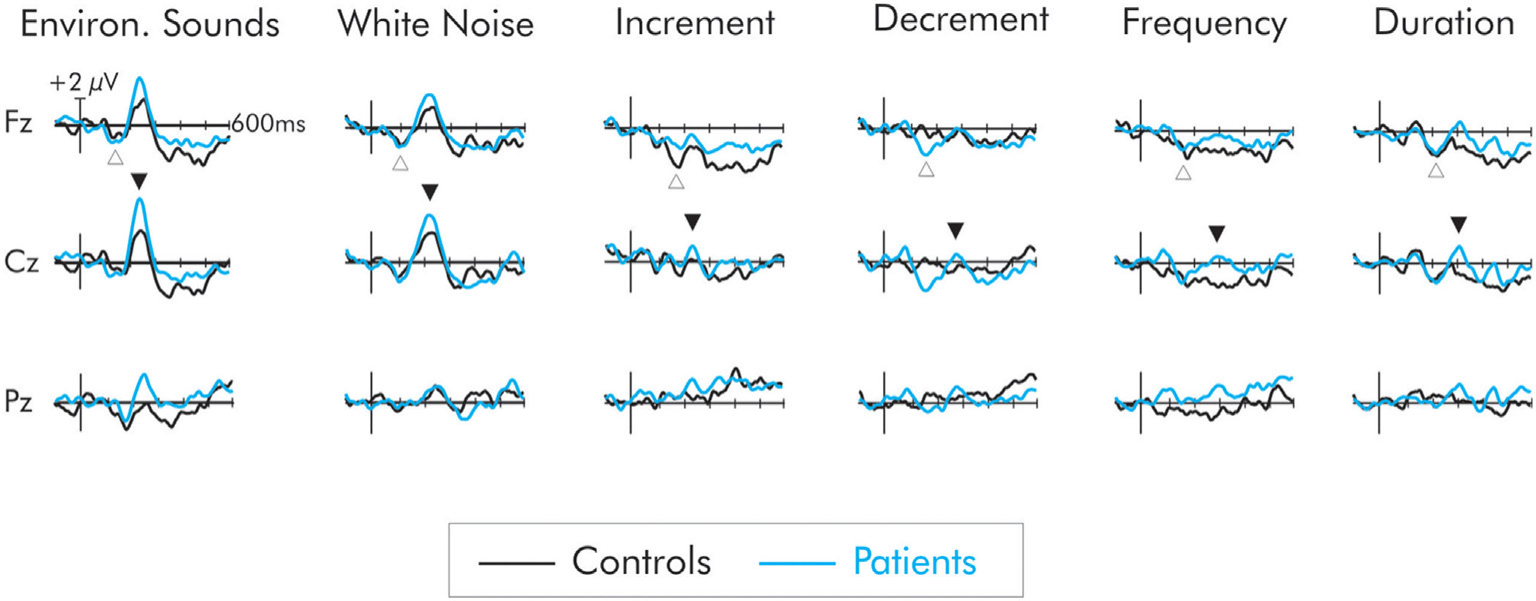
Difference waves for the six deviant stimuli for patients (blue) and controls (black). The deviant-related negativity (DRN) is indicated by an open upward arrow and the P3a by a closed downward arrow. All deviants elicited a DRN but in general, its amplitude did not differ between groups. Only the environmental sounds and white noise elicited a large P3a. The P3a for these deviants was larger in amplitude for the patient group. The deviants representing a smaller extent of change did not elicit a P3a in the control group. A small P3a was however elicited in the patients but its amplitude did not significantly differ from that of the controls.

A negativity, peaking at about 150 ms, was observed in the difference waveforms (Figure 3). In some cases, this probably reflected a larger N1 component to the deviant than to the standard. In cases in which the intensity of the deviant increased relative to the standard (i.e., increment, white noise, environmental sounds) this negativity probably reflected a composite N1 and MMN (i.e., the DRN). In the cases of the frequency, duration, and decrement deviants, the negativity probably reflects a true MMN, although for consistency will be labeled as a DRN. It was largest over frontocentral areas of the scalp and inverted in polarity at the mastoids.

A region of interest (ROI) analysis was applied to the DRN. Thus, a separate ANOVA was run on a frontal electrode (F3, Fz, F4) cluster and also on a central electrode (C3, Cz, C4) cluster where the DRN is largest. A three-way ANOVA with one between factor, group (patients vs. controls), and two within factors, deviant type (frequency, duration, decrement, increment, white noise, environmental sounds), and laterality (left, midline, right) was run. There was no significant difference in the amplitude of the DRN between patient and control groups at either the frontal or central ROIs (*F* < 1 in both cases). Similarly, there were no overall differences in the amplitude of the DRN across the deviants at frontal and central sites (*F* < 1 in both cases). The group × deviant interaction was significant for the frontal ROIs, *F*(5, 110) = 2.62, MSE = 5.22, *p* < 0.05, $\eta_{p}^{2}=0.\text{11}$. This was due to a larger DRN to the decrement deviant in patients compared to controls. The DRN did not differ as a function of hemisphere and interactions involving electrode site were not significant (*F* < 1 in all cases).

### P3a

Not all of the deviants elicited a significant P3a. In the difference waves, a large amplitude centrofrontal maximum P3a, was observed following the white noise and environmental sound deviants, peaking at about 225 and 240 ms, respectively, for both patients and controls. For the controls, confidence interval testing revealed that the P3a elicited by white noise and environmental sound deviants was significantly different from the zero amplitude baseline (*p* < 0.01 in both cases). All other deviants failed to reach significance. Similarly for the patients, only the white noise and the environmental sounds elicited a significant P3a (*p* < 0.001, in both cases). All other deviants failed to reach significance. The mean amplitudes of the P3a for both groups and all deviants are presented in Table 2.

**Table 2:** Mean amplitudes (SD in parentheses) at the Cz electrode site for the difference waves at the time interval of the P3a.

	Environmental sounds	White noise	Increment	Decrement	Frequency	Duration
Controls	1.54 (1.51)	1.82 (2.20)	−0.45 (3.22)	−0.55 (1.59)	−1.34 (2.45)	−0.35 (2.53)
Patients	4.04 (2.26)	3.32 (2.91)	0.02 (1.49)	0.07 (1.88)	0.18 (1.14)	0.88 (1.56)

An initial analysis of the data for all deviants was run at Cz where the P3a was largest. A two-way omnibus ANOVA with one between factor (groups) and one within factor (six deviants) was computed. As is apparent in Figure 3, the overall main effect of deviant was significant, *F*(5, 110) = 16.02, MSE = 3.43, *p* < 0.0001, $\eta_{p}^{2}=0.\text{42}$. Significant group differences were also found, *F*(1, 22) = 5.76, MSE = 10.72, *p* < 0.05, $\eta_{p}^{2}=0.\text{21}$. Overall, the P3a was significantly larger for the patients than the controls. The group x deviant interaction was not significant (*F* < 1).

A large P3a was only elicited by the white noise and environmental sound deviants. For this reason, a more extensive ROI analysis was carried out separately for these deviants. While the P3a tends to be largest over central regions of the scalp, its amplitude is also large at both anterior and posterior sites. Analyses were therefore run separately at frontal (F3, Fz, F4), central (C3, Cz, C4), and parietal (P3, Pz, P4) clusters. A two-way ANOVA consisting of a between factor (group), and a within factor (laterality: left, midline, right) was separately run at each cluster.

**Figure 4:**
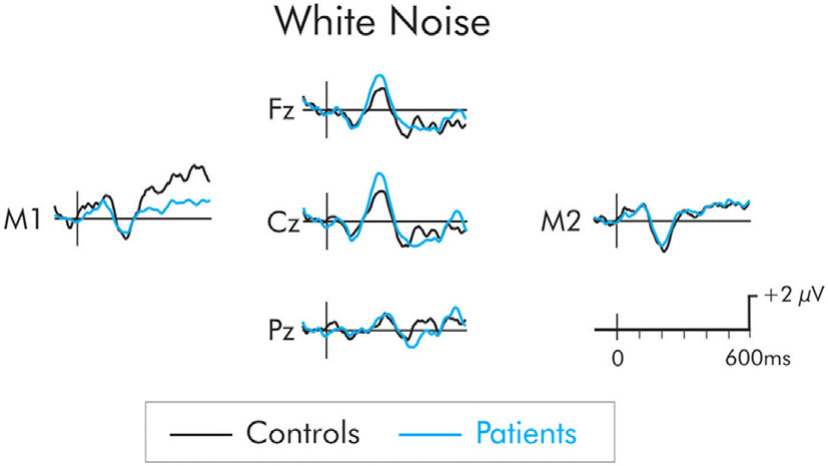
P3a following presentation of the white noise for patients (blue) and controls (black). Although, the P3a was larger in the patient group, the between group difference was not significant.

The P3a following presentation of the white noise deviant is presented in Figure 4. While the amplitude of the P3a was larger in the patients than the controls, the difference was not significant at frontal, *F*(1, 22) = 1.84, MSE = 14.06, *p* > 0.05, $\eta_{p}^{2}=0.0\text{7}$, central, *F*(1, 22) = 1.94, MSE = 16, *p* > 0.05, $\eta_{p}^{2}=0.0\text{8}$, or parietal *F*(1, 22) = 0.88, MSE = 11.46, *p* > 0.05, $\eta_{p}^{2}=0.0\text{3}$, ROIs. The P3a did not differ as a function of hemisphere and interactions involving electrode site were not significant (*F* < 1 in all cases).

**Figure 5:**
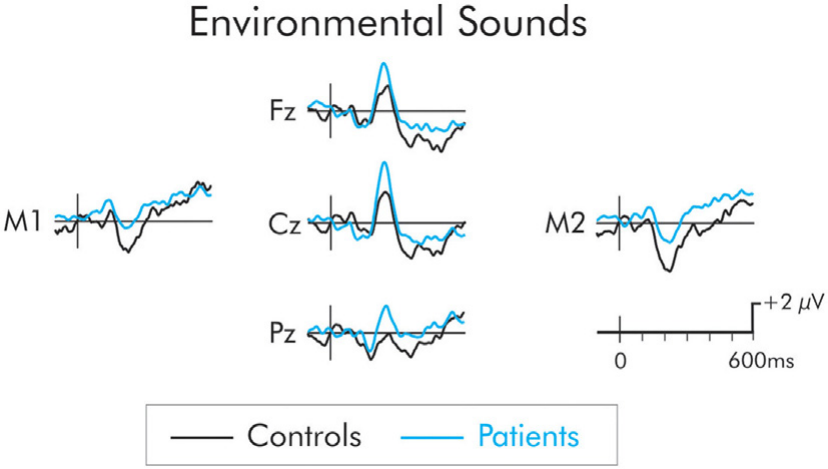
P3a following presentation of the environmental sounds for patients (blue) and controls (black). The P3a was significantly larger in patients compared to controls. The prestimulus period for the environmental sounds was not stable and varied between the two groups. As a result, a −50 to 50 ms parastimulus baseline was used.

The P3a following presentation of the environmental sound deviants is presented in Figure 5. The P3a was significantly larger in the patient group across frontal, *F*(1, 22) = 8.14, MSE = 7.51, *p* < 0.01, $\eta_{p}^{2}=0.\text{27}$, central, *F*(1, 22) = 11.32, MSE = 10.19, *p* < 0.01, $\eta_{p}^{2}=0.\text{34}$, and parietal, *F*(1, 22) = 6.29, MSE = 17.36, *p* < 0.05, $\eta_{p}^{2}=0.\text{22}$, ROIs. The P3a did not differ as a function of hemisphere and interactions involving electrode site were not significant (*F* < 1 in all cases).

The omnibus ANOVA showed the P3a was significantly larger for patients for all deviants. As is apparent in Figure 2, only a small amplitude P3a was elicited for the patients following presentation of the frequency, duration, decrement, and increment deviants. A P3a was, however, absent for the controls for these deviants. A separate group × deviant ANOVA was run only for these deviants. To maximize likelihood of finding group differences, the ANOVA was run only at Cz where the P3a was largest. The amplitude of the P3a did not significantly vary as a result of type of deviant, *F*(3, 66) = 1.00, MSE = 2.87, *p* > 0.05, $\eta_{p}^{2}=0.0\text{5}$. Importantly, while the amplitude of the small P3a was largest for the patient group, the difference compared to the control group was not significant, *F*(1, 22) = 2.51, MSE = 8.90, *p* < 0.12, $\eta_{p}^{2}=0.\text{1}0$.

Correlations were also conducted on the individual participants’ P3a amplitudes for the white noise and environmental sound deviants, and the scores on the CDI-2 and SBQ-R. Initially, these correlations were computed on all participants (patients and controls). These correlations were computed at the Cz electrode site where the P3a was largest. Correlations involving the white noise P3a were very small (*r* = −0.08 with the CDI-2 and *r* = −0.06 with the SBQ-R) and not significant. For the environmental sound P3a, the correlations were *r* = 0.48 with the CDI-2 and *r* = 0.41 for the SBQ-R, *p* < 0.05 and *p* < 0.09, respectively. Correlations with the CDI-2 subscales were also computed. The negative self-esteem, functional problems, ineffectiveness, and interpersonal problems subscales were significantly correlated with the environmental sound-P3a (*r* = 0.49, *r* = 0.57, *r* = 0.46, and *r* = 0.64, respectively). When only the patients were considered, the correlations between the amplitude of the P3a and CDI-2 and SBQ-R were much lower. The correlations between the environmental sound P3a and the overall CDI-2 and the SBQ-R were *r* = 0.05 and *r* = −0.54, respectively, and were not significant. Similarly, the correlations between the white noise P3a and each of the six subscales of the CDI-2 were also small and not significant (*p* > 0.05 in all cases).

## Discussion

A recent report by the American Centers for Disease Control and Prevention (87) has indicated that the rates for suicide have not decreased over the past several decades. This is in spite of considerably increasing mental health treatment efforts. Clearly, additional research is required to understand the factors that contribute to an increased risk of suicidal behavior.

One of these factors appears to be a dysfunction of the frontal executive functions. The present study is one of a few that have investigated an adolescent sample. This has important implications. Most previous studies have examined executive functions in adult populations. The causes and behavior associated with adolescent and adult suicide behavior may be very different. Moreover, the present study was carried out in adolescent inpatients, only days after being admitted to a local children’s hospital because of an acute risk of suicide. Most previous studies have examined non-acute suicidal ideation or those with a history of previous suicide attempts. Very few studies have studied acute suicide risk with an in-patient population. It should also be noted that several features might distinguish individuals with suicidal behavior from those who only think about suicide [e.g., suicidal ideation; (88), see also, Ref. (89)]. In their review, Bredemeier and Miller (20) indicate that a majority of studies have identified executive function deficits as a risk of suicidality, especially when associated with mood disorders. The authors emphasize individual differences. Those who have actually attempted suicide appear to show the largest executive function disorders. It is not known whether these would also differ with those manifesting an acute at risk of suicide. It is quite possible that the executive function disorders would be even larger.

Many studies have identified poorer scores in suicidal groups on a number of cognitive tasks and traditional neuropsychological tests. These however require active participation and cooperation of the individual for relatively long periods to achieve successful performance. An inability to control attention has been identified as a marker of suicide behavior (29, 32). The present study thus examined executive processes involved in the capturing of attention by a rarely occurring and potentially highly relevant but unattended stimulus input. These processes are assumed to be involuntary and operate passively, and therefore do not require active attention The frontal lobe’s central executive must make a decision regarding whether current cognitive demands or the potentially more relevant, intruding input has priority. If the rare stimulus event is given priority, then attention is switched to its processing and performance on cognitive tasks-at hand will deteriorate. There is evidence that suicidal behavior is associated with an inability to inhibit irrelevant processing. For example, individuals with previous suicide attempts have performed worse on the Stroop task, thought to reflect the ability to inhibit a dominant but inappropriate response, compared to individuals with suicidal ideation (90).

In the present study, participants were asked to watch a silent, subtitled video while ignoring the auditory stimuli. Several ERP studies have now indicated that the nature of the “diversion” task (watching the video in this case) is, in fact, relatively incidental. What the participant “is doing” should not affect the processing of the unattended auditory stimuli. These auditory stimuli consisted of a frequently occurring standard stimulus and six different rarely occurring deviant stimuli. The deviant stimuli varied in the extent to which they represented change from the standard. A series of well-studied ERP components were elicited by the deviant stimuli.

### Deviant-Related Negativity

As expected, all deviant stimuli elicited a frontocentral maximum DRN, peaking from 100 to 150 ms after stimulus onset. The amplitude of the DRN to all but the decrement deviants did not significantly differ between adolescents with suicidal behavior and healthy controls. The amplitude of the DRN was significantly larger for this decrement deviant for the patient group. It is difficult to explain why the processing of only the decrement deviant would differ. It is possible that this is a chance finding, given the large number of comparisons that were made. The decrement deviant does represent a decrease in intensity of the standard and it would be difficult to perceive. Given the fact that the patient group did display a larger P3a amplitude to all deviants, it is possible that they were able to perceive the decrease in intensity better than the controls. The general failure to find a DRN difference between the groups suggests that the automatic auditory deviance detection is well preserved in adolescents with suicidal behavior. Thus, whatever group differences were found in the amplitude of the P3a cannot be attributed to the initial detection of change. Only a limited number of studies have examined the effects of suicidality on the MMN. These studies have only used a single deviant and have focused on adult populations (91, 92). Both Zaifu et al. (91) and Zhen et al. (92) observed decreased amplitudes of the MMN in depressed adults with suicidal behavior. Studies looking at the MMN in adults with presumably only depression, also have reported a diminished MMN amplitude (93–96). The differences between these and the present study may be because of the use of different paradigms. The present study employed an optimal sequence while the others employed an oddball sequence. This is an unlikely explanation, however. Studies of the MMN have failed to find differences when it is elicited in oddball compared to optimal sequences (72, 73, 97). Importantly task demands also differed between the present study and those studying depressed adults. In the present study, the auditory MMN was elicited passively while participants watched a video. In the adult studies, participants were asked to actively attend to the auditory sequence and detect the change in the auditory stimulus. Several studies have indicated that suicidal behavioral and depression are associated with an inability to sustain attention. Differences between healthy controls and the patient group might thus be explained by this inability to sustain attention. Attention to the auditory sequence will cause other auditory ERPs to be elicited that are not observed when the auditory sequence is ignored. These attention-related ERPs may overlap and summate with the MMN. It is these attention-related ERPs rather than the MMN that might differentiate the groups. Also, processing in adult and adolescent groups may well differ. Lepistö et al. (71) studied depressed children and similar to our results, found no difference in the MMN amplitude compared to controls.

### P3a

The amplitude of the P3a was crucial to the understanding of which unattended deviant stimuli are extensively processed and interrupt executive functions maintaining attention to the task-at-hand. There is some evidence that suicidal behavior is associated with an inability to inhibit the processing of irrelevant input. In healthy young adults, a number of studies have indicated that when a pure tone is used as a standard stimulus, white noise and environmental sounds acting as deviants will be especially likely to elicit a P3a (48, 73, 98–100). So powerful are the effects of these particular deviants that they continue to elicit a P3a during the sleep onset period where conscious awareness of the external environment is gradually diminished (101). The environmental sounds even elicit a P3a during definitive stage N2 sleep (101). On the other hand, the frequency, duration, decrement, and increment deviants do not elicit a P3a in young adults during the waking state suggesting these are determined to be less relevant and thus less likely to interrupt current cognitive priorities. These results were essentially replicated in the healthy adolescent controls. A large and significant P3a was also elicited by the white noise and environmental sound deviants. No P3a was apparent to the other deviants.

To our knowledge, this is the first study to examine the P3a in suicidal adolescents. The manner in which those with an acute risk of suicide processed the different deviants was generally similar to that of the healthy controls. Thus, a large and significant P3a was observed following presentation of the white noise and environmental sound deviants. Jandl et al. (69) recorded the P3a to environmental sound deviants in depressed adults with a lifetime history of suicidal behavior. They attempted to determine whether this P3a would become attenuated over the course their study. They did not however directly present P3a amplitude differences between healthy controls and those with a history of suicidal behavior, but their Figure 2 indicates a slight enhancement of the P3a in the suicidal group. The principle finding of the present study was that the amplitude of the P3a was also enhanced in the suicidal group. When an overall ANOVA was applied to the P3a for all deviant data, a “main effect” of group was found. Thus, the P3a for all deviants was larger for those with an acute risk of suicide compared to healthy controls. The P3a was calculated in a deviant-standard difference wave. The significant differences might therefore be a result of differential processing of either the deviant or the standard. In actual fact, ERPs to the standard stimulus were not significantly different between the two groups. Thus, the enhanced P3a in the suicidal group appears to be largely due to unusual “hyper-responsivity” to the deviant. This result does need to be interpreted with caution. Even though the group x deviant interaction was not significant, follow-up testing was nevertheless deemed to be warranted. It indicated that the group P3a difference was significant for only one deviant. The P3a following presentation of the environmental sound deviant was significantly larger for the group with acute risk of suicide. The P3a to the white noise deviant did appear larger for the suicidal adolescents; however, its amplitude was not significantly different from that of controls. An intriguing finding was that a small amplitude P3a was elicited in those at risk of suicide for the frequency, duration, increment and decrement deviants while in healthy controls, a P3a to the same deviants was absent. When an ANOVA was run only on these deviants, a tendency for a larger P3a in the suicide group was apparent, although the difference again did not reach significance.

These results do therefore provide strong support for the notion that suicidal behavior is associated with deficits in attentional control and more specifically in an inability to inhibit processing of what might be irrelevant stimulus input (28). An important executive function is to determine which of the many stimulus inputs is potentially so critical to warrant an interruption of ongoing cognitive demands. A threshold set too high will result in a failure to detect truly highly relevant events, possibly critical for survival. On the other hand, a threshold set too low will result in recurrent interruption of executive functions by what turns out to be irrelevant events, resulting in frequent distraction. The later appears to be the processing option observed with suicidal behavior. Such interruptions might also explain the reported inability to maintain and sustain attention in this group. The P3a also appears to provide a measure of maturity of the frontal lobes executive function. Mahajan and McArthur (64) and Oades et al. (65) have indicated it is larger in younger than older adolescents. The finding of an enhanced P3a in the adolescents with acute risk of suicide might thus be a reflection of immature frontal executive functions. It is also tempting to generalize beyond the present findings. Suicidality has also been associated with difficulty in inhibiting negative thoughts about oneself and a tendency for mind wandering and rumination (102). In the case of the group we studied, adolescents with an acute risk of suicide, the immature frontal central executive may also have difficulty in inhibiting the urge to act on suicidal thoughts, thus the need to urgently seek help.

### Limitations

There are some limitations in the present study. The sample size is relatively small (although comparable to other ERP studies). This small sample size did not allow for a study of individual differences of other factors known to be associated with suicidality. Depression is correlated with suicidal thoughts and behavior and indeed scores on our depression index were much higher for the group at risk of suicide compared to healthy controls. Lepistö et al. (71) also reported an enhanced P3a to environmental sounds in depressed children compared to healthy controls. In our study, when the extent of depression was measured in both patients and healthy controls, a significant positive correlation was also found between this index of depression and the amplitude of the P3a to environmental sound deviants. It is therefore possible that the group differences we observed in the P3a amplitude may reflect effects of depression rather than suicidality. Keilp et al. (28, 32) noted larger impairments in executive functioning related to attention and memory in patients with high-lethality suicide attempters beyond that typically found in major depression. In the present study, when only the patients were considered, the correlation between the amplitude of P3a and depression was much lower. The extent of depression within the suicide group was thus a poor predictor of P3a amplitude and presumably its reflection of executive control of where attention is directed.

The types and dosages of various medications might also account for some of the group differences. Ideally, a non-medicated sample should also be studied. This may not be ethically or morally justifiable in those seeking emergency intervention for acute risk of suicide and deemed to require pharmaceutical treatment. Still, the types of medication used in treatment are generally considered to dampen rather than heighten the extent of information processing. It therefore seems unlikely that these medications would increase the likelihood of interruption of executive functions.

While the present study did employ an objective neurophysiological measure, the P3a, as an index of the interruption of ongoing cognitive tasks and switching of attention, it is important to note that an independent measure of this process was not available. This independent evidence of the switch of attention is generally provided by a behavioral measure, a deterioration in performance on the cognitive task in which the participant is actively engaged following presentation of the deviant. In a classic Schröger and Wolff (64) study, participants were presented with a modified oddball task consisting of short and long duration pure tones occurring with equal probability. Participants were asked to press a button corresponding to which tone had been presented. At odd times, the frequency of one of the tones was changed to form a deviant stimulus. This frequency deviation was, however, irrelevant to the primary task of duration detection and was thus to-be-ignored. The frequency deviant did, nevertheless, elicited a large P3a. Following its presentation, a prolongation of reaction times and decrease in accuracy on the duration detection task was observed. The processing of the deviant stimulus did indeed result in a switch of attention from the duration detection task resulting in the deterioration in performance. Such an active task could be modified for use with younger participants. Still, it would require the participant to remain vigilant and sustain attention for a relatively long period of time to this very difficult task. Again, attentional control appears to be highly problematic in such clinical groups.

## Conclusion

There was little evidence of a deficit in processes related to the automatic detection of acoustic change in adolescents with an acute risk of suicide. The amplitude of the DRN following presentation of the different deviants did not differ between patients and controls. Some of the deviants, particularly the white noise and the environmental sounds, were expected to interrupt executive functions that maintain attention to an ongoing cognitive task. A later positivity, the P3a, is thought to reflect processes associated with this interruption. These deviants did in fact elicit a large P3a in both groups. However, an enhanced P3a was evident in the patient group, particularly for the environmental sound deviants. For the control group, a P3a was absent following presentation of the frequency, duration, decrement, and increment deviants. These deviants did however elicit a small P3a in the group at acute risk of suicide, although the differences were not statistically significant. These findings suggest that the threshold for triggering the involuntary switch of attention might be lower in these patients and may explain their reported enhanced distractibility. This is likely to cause difficulties in concentration and may cause deterioration in academic performance, because of an inability to sustain attention. The group differences in the P3a are suggestive of an immature frontal central executive, and perhaps may provide a promising marker for the identification of those with an increased risk of suicide. This could result in an earlier recognition and, importantly, earlier intervention of suicide in adolescence.

## Ethics Statement

Written informed consent was obtained from all participants, and parents when necessary, prior to the start of the study. Participants received an honorarium for their participation. The study was approved by both the University of Ottawa’s Health Sciences and Science Research Ethics Board and the Children’s Hospital of Eastern Ontario’s Research Ethics Board. The study was conducted according to the Canadian Tri-Council guidelines (Medical, Natural, and Social Sciences) on ethical conduct involving human subjects. These guidelines are similar to those used conducted with the Declaration of Helsinki.

## Author Contributions

PT, KC, AB, AD, RR, and SG contributed to the rationale and the design of the study, and read and approved the final manuscript. The manuscript was written by PT. AB carried out the psychiatric assessment and evaluation of the patient population. RR, SG, and AB assisted with the selection of the depression and suicidal inventories. PT and AD assisted with the collection and analysis of the EEG data.

## Conflict of Interest Statement

The authors declare that the research was conducted in the absence of any commercial or financial relationships that could be construed as a potential conflict of interest.
